# An evaluation of immunization services, using the reaching every district criteria, in two districts of Gujarat, India

**DOI:** 10.1186/s41256-018-0060-4

**Published:** 2018-02-08

**Authors:** J. P. Montgomery, P. Ganguly, B. F. Carlson, N. Shrivastwa, M. L. Boulton

**Affiliations:** 1 Department of Epidemiology, University of Michigan, 1415 Washington Heights, Ann Arbor, MI 48109 USA; 2 Indian Institute of Public Health Gandhinagar, Gandhinagar-Chiloda Road, Lekawada, P.O. CRPF Camp, Gandhinagar, Gujarat 382042 India

**Keywords:** Immunization system, Evaluation, India

## Abstract

**Background:**

Immunizations are considered the most successful and cost-effective public health interventions employed today. While immunization coverage in India has improved dramatically in the last decade, areas of very low coverage persist. The University of Michigan School of Public Health and the Indian Institute of Public Health Gandhinagar collaborated to document strengths and weaknesses of immunization service delivery in two districts in India.

**Methods:**

This report describes a qualitative assessment of clinic level immunization service delivery in ten primary health centers (PHCs) located in two districts of Gujarat, India. Assessment criteria were derived from the Reaching Every District (RED) strategy that is intended to provide a framework for delivering childhood immunizations. Staff from the PHCs were interviewed in April 2013.

**Results:**

Interviews revealed several barriers to immunization service delivery including: 1) Vaccine and supply stockouts; 2) Hard to reach communities; 3) Unreliable Internet access; 4) Cold chain equipment malfunctions; 5) Inconsistently maintained and utilized immunization records and registries.

**Conclusions:**

Immunization service delivery is a complex process that can encounter barriers at many stages. A RED-based evaluation of the vaccine delivery system in Gujarat, India identified several points where the system was performing sub-optimally and possible solutions to successfully address these challenges.

## Background

Immunizations are generally considered the most successful and cost-effective public health interventions employed today [1–3]. The World Health Organization (WHO) estimates that 2 million child deaths are prevented each year through immunization, although far more could be averted through optimal use of currently existing vaccines [4, 5]. Successful immunization programs have achieved the global eradication of smallpox, elimination of polio from much of the world, and substantial reductions in illness and death attributable to diseases like measles, diphtheria, tetanus, and whooping cough.

India has made great strides in improving immunization coverage in recent years. From 2000 to 2007 estimated coverage for diphtheria, tetanus, pertussis dose 3 (DTP-3), which is commonly used as an indicator for general immunization coverage, ranged from 59 to 65% [6]. Since that time, DTP-3 coverage in India has steadily improved. In 2016, WHO estimated DTP-3 coverage to be 88% [6]. While this is a significant improvement, there are still pockets of the Indian population with significantly lower immunization coverage [7].

Gujarat, a state in the western part of India, has a population of over 60 million people and is considered to be a middle performing state with regard to immunization coverage. Gujarat reported an immunization coverage (one dose of Bacillus Calmette–Guérin (BCG) vaccine, three doses of diphtheria, pertussis, and tetanus (DPT) vaccine, three doses of oral polio vaccine (OPV) and one dose of measles containing vaccine) of 56.6% among children aged 12–23 months, according to either vaccination card or mother’s report, in 2009 [8]. The “Rapid Survey of Children” (RSOC), conducted by the Government of India (2013–14), found immunization coverage in Gujarat to be 65.3% [9]. Table 1 details the district-based immunization coverage from 2007 to 8 and 2015–16, which ranges from a high of 78.5% to a low of 30.2%. Some of these alarmingly low immunization coverage rates have persisted even as coverage has improved in India, necessitating an in depth examination of the immunization delivery system.

**Table 1:** District level immunization coverage^a^, Gujarat, India^b^

District	District level household survey 3 (2007–8) [13]	National family health survey 4 (2015–16) [14]
Dohad	32.9%	33.0%
BanasKantha	38.9%	35.3%
The Dangs	39.3%	44.3%
PanchMahals	46.1%	30.2%
SabarKantha	47.6%	49.1%
Surendranagar	49.0%	37.5%
Kachchh	49.2%	45.0%
Amreli	50.5%	59.9%
Valsad	51.8%	52.9%
Ahmedabad	53.7%	49.0%
Kheda	54.1%	39.5%
Jamnagar	56.4%	71.4%
Bharuch	56.8%	56.9%
Bhavnagar	57.4%	52.4%
Vadodara	59.6%	63.3%
Rajkot	62.3%	51.4%
Narmada	64.3%	69.3%
Gandhinagar	65.2%	66.1%
Junagarh	66.7%	56.5%
Anand	68.8%	61.4%
Patan	70.2%	30.7%
Mehsana	72.0%	55.1%
Navsari	74.0%	78.5%
Porbandar	76.7%	68.8%
Surat	88.2%	48.0%
Tapi	n/a	72.9%

^a^Children age 12–23 months who have received one dose of Bacillus Calmette–Guérin vaccine (BCG), three doses diphtheria, pertussis, and tetanus vaccine (DPT), three doses polio vaccine, one dose of measles-containing vaccine

^b^Study selection was based on DLHS-3 data. NFHS data are included here for comparison purposes but were not available at the time of the study

In 2002, the Reaching Every District (RED) strategy was developed and introduced by WHO, the United Nations Children’s Fund (UNICEF), and other partner agencies in Gavi, the Vaccine Alliance in order to improve immunization systems in areas with low coverage [10]. The strategy outlines five operational components that are specifically aimed at improving vaccination coverage in every district: 1) Planning and management of resources; 2) Re-establishment of regular outreach services; 3) Community links with service delivery; 4) Supportive supervision: on-site training; 5) Monitoring and use of data for action. Each of the categories includes a list of indicators for evaluating and implementing optimal immunization programs.

This article describes qualitative research, conducted in 2013, that may help to guide ongoing efforts to improve immunization coverage for some areas of India that have experienced persistently low coverage levels. The University of Michigan School of Public Health (UMSPH) and the Public Health Foundation of India conceived and planned this study and the UMSPH and Indian Institute of Public Health Gandhinagar (IIPHG) collaborated to conduct the study utilizing the RED indicators to assess strengths and weaknesses of immunization services in select PHCs located in Gujarat, India. The study was reviewed and classified as “not regulated” by the University of Michigan Institutional Review Board; the IIPHG Institutional Review Board accepted this classification. All necessary government approvals were obtained.

## Methods

### Site selection

Data from the District Level Household Survey 3 (DLHS-3) were used to identify immunization coverage in the 25 districts within Gujarat with relatively high levels of immunization coverage and districts with low levels of immunization coverage (Table 1). Mehsana (population 2 million, immunization coverage 72%) was selected as a district with high level of immunization coverage, and Dahod (population 2.1 million, immunization coverage 32.9%) was selected as a district with significantly lower levels of immunization coverage. While other districts also met our selection criteria, these districts were selected due to relatively convenient proximity to IIPHG. In each district, 7 primary health centers (PHCs) were selected based on a random selection of blocks within each district (for a total of 14 PHCs; Dahod had 65 PHCs and Mehsana had 55 PHCs). A random selection of five of the seven selected PHCs in each district were approached for participation. The remaining two PHCs were held in reserve in case of refusal. No PHC refused the initial recruitment request.

Districts were selected based on DLHS 3 data and a random selection of blocks.

### Questionnaire

The questionnaire for assessing immunization delivery included six sections, one for each of the five RED operational components and a sixth section based specifically on cold chain issues; problems in the cold chain were identified by IIPHG as being potential barriers to effective immunization delivery in India. Each section included a list of dichotomous questions/statements that were based on the RED criteria for each specific operational component and open-ended questions that aimed to identify barriers to optimal vaccine delivery and facilitators for overcoming these barriers.

### Data collection

Professional staff from 10 PHCs were interviewed in one group interview per PHC. Interview participants included the PHC medical officer, auxiliary nurse midwife (also known as Female Health Worker in some areas of India), the accredited social health activist (ASHA), the person responsible for maintaining vaccine cold chain, local health officials, and others deemed appropriate by the individual PHC staff. Interviews lasted approximately 2 h and were conducted in Gujarati, which is the local language. Questionnaires were developed in English and then translated and back translated by research team members who are fluent in both languages. All interviews were conducted in April 2013 by the same interview team, which included researchers from UMSPH and IIPHG. The team was trained to conduct these specific interviews.

### Data analysis

**Fig. 1:**
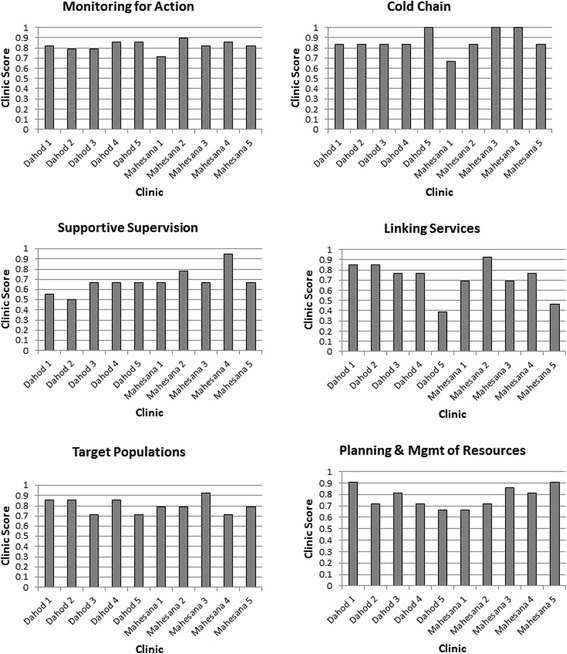
Comparison of index-based scores across 10 PHCs in Gujarat, India

We created six indices corresponding to each of the questionnaire sections, and assessed proportion scores (range 0.0–1.0) based on the number of criteria that a PHC met. Each index included a different number of criteria questions: 1) Planning and management of resources included 21 questions, 2) Re-establishment of regular outreach services included 14 questions, 3) Community links with service delivery included 13 questions, 4) Supportive supervision included 18 questions, 5) Monitoring for and use of data for action included 28 questions, and 6) Cold chain monitoring included 6 questions. A PHC that met all criteria in a single category received a score of 1.0 for that category. Scores were then mapped to bar graphs for visual comparison to identify general strengths and weaknesses by PHC and category (Fig. 1).

We further examined specific weaknesses by looking at the responses to both the scaled questions and the open-ended questions. The analyses were done at the district level to identify themes while maintaining PHC confidentiality. Frequencies and theme-based summaries are reported below.

Data were analyzed using SAS software, version 9.3. Copyright © 2011, SAS Institute Inc. SAS and all other SAS Institute Inc. product or service names are registered trademarks or trademarks of SAS Institute Inc., Cary, NC, USA.

## Results

Figure 1 illustrates the score that each PHC received (scale 0.0–1.0) for each of the six indices. Scores ranged from a low of 0.38 from one PHC in Dahod that only met 38% of the “community links with service delivery” criteria, to a high of 1.0, from 3 PHCs (1 PHC in Dahod and 2 PHCs in Mehsana) that met 100% of the “cold chain monitoring” criteria. “Monitoring and use of data for action” had the least variability across PHC sites (range 0.71 to 0.89), followed closely by scores for “re-establishment of regular outreach services” (range 0.71–0.93) and “planning and management of resources” (range 0.67–0.90). Scores for the other three indices had greater levels of PHC-based variation: “cold chain monitoring” (range 0.67–1.0), “supportive supervision” (range 0.5 to 0.94), and “community links with service delivery” (range 0.39–0.92). Dahod, the low immunization coverage district, generally had lower index scores than Mehsana, but this was not consistent for all PHCs nor for all indices.

In order to best describe the immunization delivery problems, we further examined the individual questions in each index.

### Planning and Management of Resources

Four of the PHC informant groups indicated that general immunization activities were compromised due to funding shortfalls (3 PHCs in Dahod, 1 PHC in Mehsana). Stock outs of vaccines and other related supplies were identified as problems in many PHCs (4 PHCs in Dahod, 5 PHCs in Mehsana). Specifically, PHC informants indicated an inadequate supply at the time of the interview of the following: vaccines (3 PHCs in Dahod, 4 PHCs in Mehsana); safe injection equipment (1 PHC in Dahod, 1 PHC in Mehsana); fuel for vehicles (1 PHC in Dahod, 1 PHC in Mehsana); and other supplies related to vaccine delivery (3 PHCs in Dahod, 5 PHCs in Mehsana). In general, vaccine stock-related issues were problems for both Dahod and Mehsana.

Staff retention and long-term job vacancies were identified as barriers to providing consistent vaccine delivery (5 PHCs in Dahod, 3 PHCs in Mehsana). The open-ended questions identified a need for programmatic emphasis on hiring new staff as positions become available, and there was an expressed desire to receive support for this from district health officials. Since this issue came out of the qualitative questions, we were unable to determine if the remaining 2 sites in Mehsana had similar problems.

### Re-establishment of regular outreach services

During vaccination sessions, six PHCs did not record dose-information in an electronic immunization registry (5 PHCs in Dahod, 1 PHC in Mehsana). Hand-written tally sheets were used by eight PHCs to keep track of vaccinations given (4 PHCs in Dahod, 4 PHCs in Mehsana). Interviewees mentioned that patients often did not have their government-issued immunization cards available during visits, which may make it difficult for immunization providers to determine which vaccines are needed during the visit. We did not assess if these cards were forgotten, lost, destroyed, or originally given to patients.

Interviewees discussed the challenges associated with delivering vaccine to various populations because of socio-demographic, geographic, and cultural barriers. The migrant population, in particular, was identified as a specific sub-population that is notoriously difficult to reach with immunization services. Staff from one PHC in Dahod suggested targeting these populations during the marriage season and during festivals since these represent events where migrant population traditionally gather. It is also a time when people go to their family home, potentially making it easier to find them, compared to other times during the year.

### Community links with service delivery

Community leaders can be tremendously useful to health care providers, especially when it comes to improving vaccination coverage. In both Mehsana and Dahod, community leaders were involved in improving immunization delivery. Most PHCs included community leaders in their advisory committees (4 PHCs in Dahod, 4 PHCs in Mehsana). In Dahod, four PHCs did not include community leaders in the planning process specifically for immunization delivery, but all five PHCs in Mehsana included them in this process. Community leaders often identified populations in need of vaccine (4 PHCs in Dahod, 3 PHCs in Mehsana), secured venues for outreach PHCs (4 PHCs in Dahod, 2 PHCs in Mehsana), identified barriers to vaccination in a community (4 PHCs in Dahod, 3 PHCs in Mehsana) and provided vaccine and VPD-related health education to the community (4 PHCs in Dahod, 4 PHCs in Mehsana). Fewer than half of the PHCs had educational material for community leaders (1 PHC in Dahod, 3 PHCs in Mehsana), but many held information sessions for the community leaders to educate them about vaccination and vaccine-preventable diseases (3 PHCs in Dahod, 3 PHCs in Mehsana). Community leaders helped to identify newborns (4 PHCs in Dahod, 3 PHCs in Mehsana) and pregnant women (4 PHCs in Dahod, 3 PHCs in Mehsana). Community volunteers were used to help during immunization sessions at all sites.

### Supportive supervision

Interviewees stated that the supervisors were qualified to provide supervision (all 10 PHCs), they established assessment tools to identify problems (all 10 PHCs), and most of them spent time in understanding barriers to vaccine delivery (4 PHCs in Dahod, 5 PHCs in Mehsana). Most supervisors offered guidance in overcoming these barriers (4 PHCs in Dahod, 5 PHCs in Mehsana), and PHC informants generally viewed the guidance as helpful (4 PHCs in Dahod, 5 PHCs in Mehsana). Many PHC informant groups reported that they would like to have more supervisory visits (4 PHCs in Dahod, 2 PHCs in Mehsana).

### Monitoring and use of data for action

All PHC informants reported that they used data to inform immunization activities; most created monitoring charts to track immunization coverage (3 PHCs in Dahod, 4 PHCs in Mehsana). Most PHC informants reported that an electronic immunization registry was in use (4 PHCs in Dahod, 4 PHCs in Mehsana) and worked well (4 PHCs in Dahod, 4 PHCs in Mehsana). All PHC informants reported that they used paper-based systems for tracking immunizations and all reported that this type of system worked well. However, qualitative reports indicate that hardcopy immunization records were often not available during immunization sessions.

Qualitative reports stated that electricity and Internet access were not consistently available. This leads to multiple potential problems, including making immunization workers unable to reliably access electronic immunization registries. While Internet access is an issue common to many areas of India, availability of electricity in Gujarat is considered to be some of the best in the country. Some of the staff at PHCs in Dahod mentioned frequent power (electricity) cuts, but we could not get specific information of duration of such interruptions. Power cuts were not mentioned in Mehsana. With the available information, we cannot conclusively gauge the extent to which power outages were a bottleneck.

### Cold chain monitoring

Adequate equipment to maintain vaccines at the temperature required for maximum efficacy (cold chain equipment) is a crucial part of an effective vaccine delivery system [11]. While most PHCs reported having adequate staffing (9 PHCs) and equipment (all 10 PHCs) to maintain the cold chain of vaccines, only three PHCs (1 PHC in Dahod, 2 PHCs in Mehsana) stated that their cold chain equipment works all of the time. Furthermore, one PHC informant group in Mehsana stated that it rarely worked. Our data show that six PHCs mentioned some cold chain equipment malfunction, but we don’t have specific data as to the duration of the problem and the specific type of equipment that did not work.

## Discussion

Immunization service delivery is a multifaceted and complex process that can break down at many stages, contributing to low immunization coverage and a higher burden of morbidity and mortality due to vaccine-preventable disease in children, particularly in a developing and highly populated country such as India. A RED-based evaluation of the vaccine delivery system in two districts in Gujarat, India identified several points where the system for immunization service delivery needs further strengthening. The major issues that emerged from this evaluation included inconsistent availability of supplies, inconsistent electricity, inconsistent access to the current paper-based immunization records system during immunization sessions, difficulty in reaching migrant populations, and cold chain equipment malfunctions in some cases.

Some of the issues that we identified may be beyond the control of the PHC level and need to be addressed at the district, state and even national level. Many PHCs reported problems with consistently stocking vaccines and related supplies, and had difficulty maintaining consistent and adequately trained staff. Stocking and staffing issues likely impact PHCs throughout the state and perhaps throughout India.

Another major factor usually found to limit a PHC’s ability to provide immunization services is limited or inconsistent electricity. Electricity is considered to be quite good in Gujarat, but was mentioned as a problem in some of the PHCs. This may have far reaching implications that compound other service delivery challenges such as cold chain equipment and electronic immunization records. Cold chain equipment malfunctions, for any reason, may place PHCs in a situation of delivering vaccine that has not been properly stored and may therefore be rendered ineffective. However, ice lined refrigerators (ILRs) can maintain a stipulated temperature for extended periods of time and are often provided as a short-term solution to temperature maintenance problems. ILRs were present in all the health centers.

If all of India is to see significant improvements in childhood vaccination coverage, it will be necessary to develop methods for consistent provider-level access to immunization records, and ideally an electronic immunization registry, accessible during every immunization delivery session for every child regardless of where previous vaccines may have been received. Most of the PHCs in this study used an electronic registry but relied heavily on hard copy patient immunization records that were often not available during immunization clinics. India’s system of paper documentation for childhood vaccination that is not always available on site, coupled with unreliable electricity, reduces access to important information and increases the likelihood of missed opportunities for vaccination. The Maternal Child Tracking System was launched by the Indian Ministry of Health to assist with this problem [12]. Unfortunately, this system is not accessible without electricity, and not usable without properly trained data entry staff. While PHCs reported using electronic registries, there are currently major limitations to these. Devices such as hand-held tablets with Wi-Fi hotspot and/or cellular technology may be suggested to overcome some of these barriers and would allow instant access to a registry, thus having the most current information about needed vaccines readily available.

To improve vaccine coverage among migrant communities and other hard to reach populations, one innovative suggestion from a participant was to implement targeted immunization programs during the marriage season and during festivals. Because people travel to family homes for these events, it may be easier to reach them compared to other times during the year. Perhaps there should be additional or special vaccination outreach activities during these events. This strategy may be a viable option to reach unimmunized groups, but needs to be supported with adequate supplies and a robust tracking system because all doses of childhood immunization cannot be fully completed during a single period of time.

This qualitative study is obviously limited by its size. However, the depth of research and responses identify clear issues that need to be quantified and improved upon in order to increase immunization coverage in Gujarat and other areas of India. Additional information collected directly from clients and from direct checks of the facility would add to the depth of this study, and would be useful next steps.

## Conclusions

The goal of this study was to use established criteria to identify weaknesses and to understand differences in vaccine delivery system implementation at the PHC level, between a high and a low immunization coverage district in Gujarat, India. We expected to see serious breakdowns in the vaccine delivery system, particularly in Dahod (the low coverage district). However, while PHCs did not report optimal vaccine delivery (based on the RED criteria) they reported that overall they have very high coverage within their jurisdiction and identified very few problems. This in itself is inconsistent as immunization coverage in both districts is clearly suboptimal, based on estimates of immunization coverage in each district and the epidemiology of many childhood vaccine preventable diseases that are still endemic or epidemic in India. These issues must be addressed in order to improve vaccination coverage of India’s children, particularly as additional vaccines are added to India’s National Immunization Schedule. A robust vaccine delivery system is needed in India to insure age-appropriate immunization of all children and to realize a significant reduction in the incidence of vaccine-preventable diseases.

## Abbreviations

ASHA: Accredited Social Health Activist
BCG: Bacillus Calmette–Guérin vaccine
DLHS: District Level Household Survey
DPT: diphtheria, pertussis, and tetanus vaccine
IIPH: Indian Institute of Public Health
ILR: Ice Lined Refrigerators
NFHS: National Family Health Survey
PHC: Primary Health Centers
PHFI: Public Health Foundation of India
RED: Reaching Every District
UMSPH: University of Michigan School of Public Health
UNICEF: United Nations Children’s Fund
WHO: World Health Organization
